# Perceived Safety and Pedestrian Performance in Pedestrian Priority Streets (PPSs) in Seoul, Korea: A Virtual Reality Experiment and Trace Mapping

**DOI:** 10.3390/ijerph18052501

**Published:** 2021-03-03

**Authors:** Haeryung Lee, Seung-Nam Kim

**Affiliations:** 1 Architecture & Urban Research Institute, Sejong 30103, Korea; hr100425@gmail.com; 2 Department of Urban Design and Studies, Chung-Ang University, Seoul 06974, Korea

**Keywords:** pedestrian priority street, shared space, pedestrian performance, perceived safety, walking environment, virtual reality

## Abstract

Pedestrian Priority Street (PPS) project, launched to encourage safer and more convenient walking by improving the inferior pedestrian environment on narrow streets without sidewalks, is based on Monderman’s shared space concept. Similar to the shared space approach, PPS aims for mutual consideration between pedestrians and drivers and strives to create a pedestrian-friendly environment, but the project relies on a unique road surface design. Considering the two main goals of the PPS project, this study investigated how subjective safety and pedestrians’ movements differed by design types. To analyze safety perception, ordered Logit regression and post-hoc interviews were conducted with visual assessment survey using recorded VR (virtual reality) videos. Next, trace mapping and analysis were performed based on the video recordings to measure the degree of free walking. The results found that pedestrians perceived higher safety level in PPSs than in general back road. Further, the pedestrians moved more freely in the street with an integrated design. In other types, which suggested a pedestrian zone at the roadside, there was not much difference in behavior from the general back roads. Thus, the design principle of PPS, which does not set a boundary between pedestrian and vehicle area, should be observed to lead to behavioral changes in pedestrians.

## 1. Introduction

A narrow street without sidewalks is representative of spaces that threatening pedestrian safety in cities. These streets, called alleys, back roads, or access streets, are found all over the world including in rapidly developing megacities where infrastructure cannot keep pace with economic growth as well as in old towns in advanced countries where organic patterns remain [1]. In such streets, pedestrians are forced to share the spaces with vehicles under extremely dangerous conditions [1].

Seoul, South Korea is no exception to this problem. Its traditional urban tissue includes many narrow streets without sidewalks, named *i-myeon-do-ro* (literally “back road”). In terms of total length, *i-myeon-do-ro* less than 12 m wide (hereafter, just “back roads”) comprise about 76.8% of the streets in Seoul by length [2], and about 66.9% of pedestrian traffic accidents occurred on these streets in Korea [3]. It is hard to be sure that installing narrow sidewalks on these roads is an optimal alternative for pedestrians. Due to the characteristics of back roads, which are used for direct access to buildings, pedestrians use these roads frequently. If pedestrians and vehicles are completely separated by narrow sidewalks, dangerous situations could occur when pedestrians are driven out of the sidewalk. Otherwise, they must endure the limited space and inconvenience of walking.

To enhance pedestrian safety and right in these streets, the Seoul city government has implemented the Pedestrian Priority Street (PPS) project since 2013. This project is conceptually rooted in the shared space approach of Hans Monderman [4,5]; but it is quite distinctive in comparison with well-known shared street projects worldwide, such as Exhibition Road in London. As a representative example of shared space, Exhibition Road is wide enough to ensure a safe pedestrian zone while also creating an integrated space by applying a “single surface” treatment across the entire width of the street [6] (p. 7). However, this project targets only inferior roads where pedestrians and cars merge due to their narrow width (less than 10 m). In such a small space, the extent to which changes can be mad—such as installing sidewalks, street facilities, and trees—is restricted. Accordingly, this project mainly relies on the pavement surface design including geometric patterns and colors usually employed in the sidewalks. Through this, this project aims to ensure pedestrian safety while allowing them to recognize and utilize the entire road as a pedestrian space rather than vehicle space.

However, opinions are divided on the effectiveness of these design techniques, and empirical evidence is still insufficient. Kim and Shim [7] verified the impact of surface design on driving behavior through driving experiments and determined that the visual elements of PPS were insufficient to cause decisive changes in driving. However, Lee and Kim [1], who analyzed the vehicle speed measured before and after the PPS project, argued that applying inappropriate design would rather increase speed. They also suggested that pedestrians’ perceived risk could vary depending on the environment. In particular, they indicated the requirement to discuss the difference between a design with hints of a pedestrian zone and a design that is completely integrated, but an accurate verification was not made. Similarly, Namgung and Park investigated the speed and deceleration factors in PPS and pointed out the difficulty of determining the effectiveness of the project merely by measuring the speed change [8]. Meanwhile, Kim and Lee measured the effectiveness of the PPS project with a traffic accident analysis and observed a decrease in the number of traffic accidents after the project [9].

Most previous studies have mainly evaluated the safety in PPS using objectified evidence such as traffic accidents and vehicle speeds. Even though Kim and Shim analyzed the impact of microscopic design elements on road users, this experiment was conducted only on drivers [7]. The most accident-prone road users are pedestrians on the back roads, and it is important to investigate whether pedestrians perceive themselves as safe. In fact, judging pedestrians’ safety by the number of accidents has the potential risk to underestimate the danger of the accident [10]. Schneider et al. revealed a discrepancy between the actual frequency of traffic accidents and pedestrians’ perceived danger [10]. Moreover, it is also necessary to pay attention to pedestrian behavior, because the PPS project aims to allow pedestrians to use the full width of the road more comfortably than before in addition to enhancing pedestrian safety. However, little is known about pedestrian movements in PPS, which is an important factor indicating how freely pedestrians use the whole area of the road.

In this context, this study examined the effectiveness of the PPS project in terms of pedestrians’ perceived safety and pedestrian behavior. We analyzed the influences of each type of paving design on changes in subjective safety and walking patterns. First, to investigate perceived safety in PPS, a visual assessment survey was conducted using recorded virtual reality (VR) videos. Recent studies using VR have dealt with perception and cognition related to the urban environments [11,12,13,14], and VR has been widely acknowledged as a research instrument [15,16,17]. In particular, VR could be used as a means to investigate the risk perception of pedestrians. Llinares et al. used VR in an experiment to examine the influence of street design on pedestrian’s perceived safety [14]. Yet et al. also conducted an VR-based study of pedestrians’ risk-taking behavior [18]. Unlike these studies, which used simulated VR to generate non-existence spaces, recorded VR was employed in this study. Recorded VR is an effective alternative to creating realistic environment [19]. Mouratidis and Hassan argued that a 360 VR video is valuable than traditional images when evaluating users’ perceptions of environments [20]. Moreover, this makes it possible to perform an experiment—which cannot be carried out in the real field due to safety issues—by recording the produced dangerous situation and allowing participants to experience it as if they are in the field. Next, we analyzed video recordings and performed trace mapping analysis in order to see if pedestrians freely use the entire space of the street. This analysis method has been widely used to examine pedestrian behavior in previous studies. Mapping crossing points and routes has been used as a tool for evaluating pedestrian’s comfort and traffic quality in shared spaces [21,22,23]. The task of tracing and recording all the walking path directly allows us to determine the change in walking behavior by creating shared space [23,24]. In this way, the results of observing, recording, and quantifying pedestrians are meaningful in itself when analyzing pedestrian behavior. Taking these into consideration, we would like to verify the effectiveness of PPS and, based on the results, propose the proper direction of surface design for a more successful operation of PPS project. This is expected to help solve issues of pedestrian safety in many other cities that have the similar street environments.

## 2. Materials and Methods

### 2.1. Study Area

**Figure 1:**
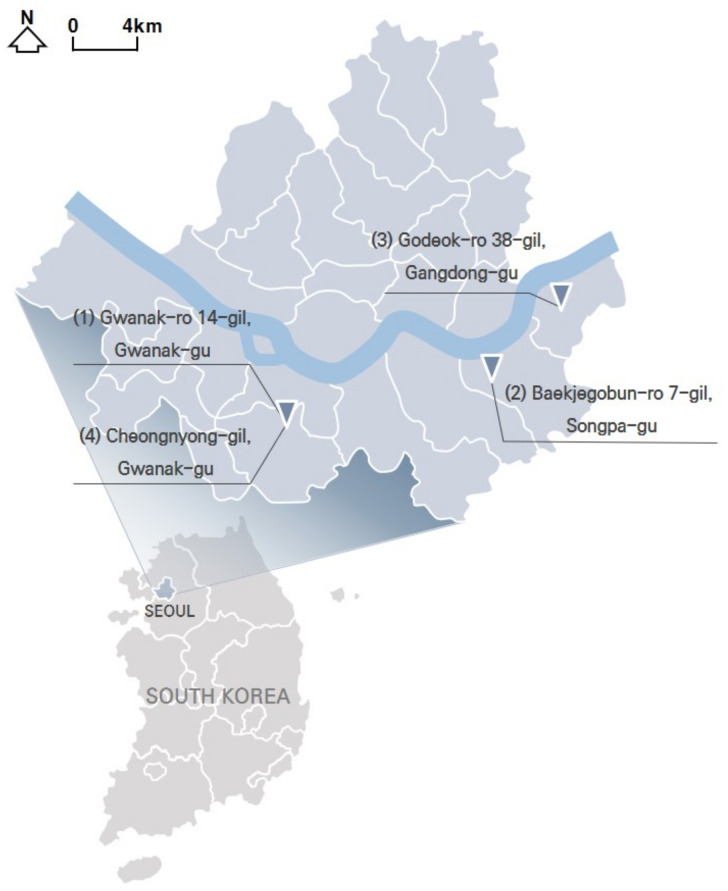
Distribution of the study sites.

To test distinctive effects by design type, among the PPSs completed in 2014–2017, we selected three representative streets based on Lee and Kim’s standard [1] (pp. 6–7). They classified the PPSs into three type depending on “the extent of “visual separation (VS)” between vehicles and pedestrians, which was created by the paving patterns” [1] (p. 6; Table 1). In addition, one typical street in Seoul that has similar conditions with PPS apart from pavement design was selected for a control group. Figure 1 and Table 2 illustrate key characteristics of the selected study sites.

Gwanak-ro 14-gil (type A) is a narrow one-way road, only 5-meters wide. The continuous decussate pavement pattern was diagonally applied to the full width of the road. Baekjegobun-ro 7-gil (type B) is an 8-meter-wide two-way road. Repeated transversal lines were employed, but only for the central part of the road. On the roadside edges, colors contrasting with those of a typical road were painted in line with the driving direction of vehicles, as if it were a walkway. Godeok-ro 38-gil (type C) is a wider road compared to the others. Typical asphalt pavement in the central part was retained and stamped asphalt pavement was applied only on the roadside areas. Thus, it seems to strictly limit pedestrian movement to the edge of the road. The 7-meter-wide Cheongnyong-gil is a typical back road without sidewalks in Korea.

### 2.2. Analytical Overview

This study analyzes the effects of PPS projects from two perspectives. First, we examine pedestrians’ perceived safety in PPSs through an ordered logit analysis using the data from a visual assessment survey with omnidirectional (360°) videos viewed with head-mounted VR device (hereafter, “omnidirectional video-based VR” or just “360 VR video”). We also conduct in-depth interviews for an exact interpretation of the statistical analysis. Second, we determine pedestrians’ degree of free walking in PPSs by employing various indicators such as “curvature of walking path”, “proportion of walking trace in central part of the street”, and “informal crossing ratio”. To this end, we record pedestrian behaviors using video cameras and apply a tracing method. We then quantitatively analyze their walking behaviors by street type using GIS techniques and *t*-test.

### 2.3. VR Experiment for Analyzing Perceived Safety in PPSs by Design Type

#### 2.3.1. Experiment Design and Omnidirectional Video Recording

**Figure 2:**
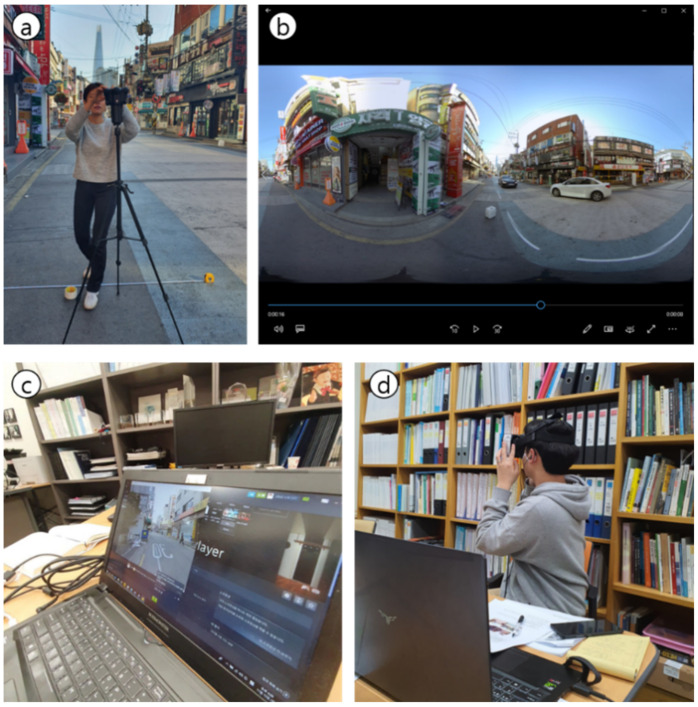
Omnidirectional video recording and experiment setting. (**a**) omnidirectional camera setting; (**b**) an example of edited 360-degree video (captured image); (**c**) real-time monitoring screen of the survey using HMD; (**d**) actual VR experiment scene.

To evaluate pedestrian’s perceived safety level in PPSs and compare it with that of general back roads, we conducted a controlled VR experiment (Figure 2). The experiment aims in particular to compare the degree of pedestrians’ perceived safety by street type when they are standing on the roadside when a vehicle passes. The specific experiment setting is as follows. (1) The directed scenario for the experiment introduces a typical dangerous condition in back roads when a vehicle passes by a pedestrian standing on the roadsides with a 1-meter gap. (2) In place of pedestrians, an omnidirectional camera (Kandao Obsidian R) (KanDao Technology Co., Ltd., Shenzhen, China) with a lens height of 170 cm was installed on the roadside area. It ensures the safety of the experiment and at the same time accurately conveys the danger felt by pedestrians on the road to other survey participants. (3) The vehicle speed was fixed at 30 km/h and was driven by the author. For accurate driving, we marked a movement line in advance on the road. We filed all these experiments conducted in four study sites from 6 AM to 12 PM on Saturday, 19 October 2019. In order to prevent interference by vehicles other than the designated vehicle in advance, the experiment and filming was carried out during the morning hours of the weekend when there was little traffic. For convenience of the visual assessment survey, the recorded videos were edited into a 20-s video clip for each street.

The research protocol and survey instruments were approved by the IRBs at Chung-Ang University, and all study participants provided informed consent.

#### 2.3.2. Visual Assessment Survey

A visual assessment survey using HMD was conducted from 21 to 22 November 2019 in order to compare the degree of safety felt in 360-degree videos taken on each street. A total of 50 participants were recruited using a snowball sampling process, starting with students from the Department of Urban Design and Studies at Chung-Ang University. The target population and sampling method were decided by considering the street visitors’ main age group (20s and 30s) and convenience of the survey process using VR equipment. Table 3 shows the basic characteristics of the participants.

Based on Rundmo and Iversen [25], we asked the participants to view the recorded VR video and score the level of risk perception in each street via a 7-point Likert scale. Existing studies have generally used rating scales with five to seven points [26,27]. Considering the fact that most of the participants in this study had related majors and that the number of samples was relatively small due to the nature of VR experiments, we chose a 7-point scale for more detailed differences in levels. For consistency with the following analysis, the answers were reverse coded ranging from “(1) *very dangerous: significant danger of colliding with the vehicle*” to “(7) very safe: *no possibility of collision with the vehicle*”.

#### 2.3.3. Ordered Logit Analysis

To examine the impact of street design type on pedestrians’ perceived safety, we employed ordered logit analysis. Perceived safety level, as measured on the 7-point Likert scale, was applied as a dependent variable. Here, participants’ responses to the four streets were considered separate samples; therefore, the total number of observations is 200 in the model. Our test variables are defined as three dummy variables for pavement design type (i.e., types A, B, and C), and a general back road without design pavement functions as the reference group in the model. We controlled for personal experiences and attitudes related to traffic accidents or safety in back roads: Whether they have a driver’s license, a car, car accident experience (their own or an acquaintance’s), attitude to speed limits (30 km/h), and frequency of walking in back roads. Other control variables include previous VR experience, major, and number of years of education.

#### 2.3.4. *Post-Hoc* Interview and Content Analysis

After carrying out the visual assessment survey using recorded VR, in-depth interviews were conducted to properly understand how the participants felt about the experience in each street. The main questions included (1) specific reasons for their risk perception level, (2) whether the level of risk perception had changed depending on the street width, (3) preference for the separation of pedestrian and vehicle areas in back roads, and (4) opinions on the PPS project and its design styles. We analyzed interview contents and classified and counted main opinions. This analysis result can contribute to a more accurate interpretation of the results of the ordered logit analysis.

### 2.4. Trace Mapping for Analyzing the Degree of Free Walking in PPSs by Design Type

#### 2.4.1. Video Recording of Pedestrian Movements

To examine pedestrians’ degree of free walking in PPSs by design type, we applied trace mapping methods. This analysis aimed to verify whether the original purpose of the PPS project, which was intended to encourage pedestrians to freely walk through the entire space including the central part of the road, has been achieved. To this end, we filmed four streets simultaneously from 7 a.m. to 9 p.m. on Wednesday, 6 December 2019. Two straight segments (about 30 m long) were selected for each street, and cameras were installed in a two to three-story building overlooking the road so that pedestrians’ behavior and the road pavement design could be well identified.

We checked all the recording files (14 h × 8 cameras) and identified three peak periods: commuting time (08:00–09:00), after-lunch time (13:00–14:00), and early evening (17:00–18:00). Among them, a section of 15 min (13:30–13:45)—when there was no reflection of light from the camera and the shade was similar at all study sites—was selected for detailed trace mapping analysis. In this process, we also considered that cameras’ view at all streets should not be obscured by obstacles, while simultaneously, moderate conflicts between pedestrians and vehicles should occur.

#### 2.4.2. Pedestrian Movement Tracing and Analysis

**Figure 3:**
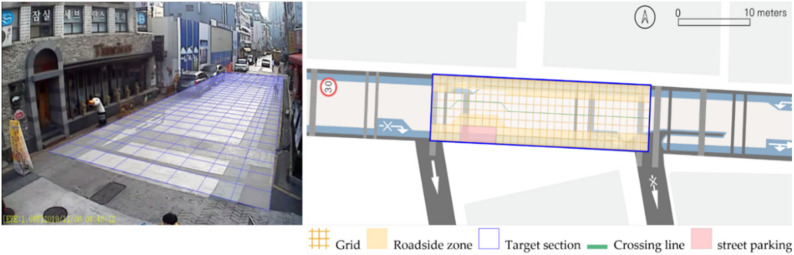
Example of grid projection and defining roadside zone and centerline (Baekjegobun-ro 7-gil).

To quantitatively measure the degree of free walking in PPSs, we applied the tracing method. Gehl regarded this approach as an efficient behavior observation and analysis technique [28] (p. 28). We tracked and digitized the walking paths of all pedestrians (a total of 305) appearing in the extracted video clips. For efficient and accurate analysis, a grid network of 1 m × 1 m cells was projected onto the video screen (Figure 3). Interrupted walking, such as entering a nearby building or boarding a vehicle, was excluded from the analysis. When several pedestrians moved in a group, only one path was created based on the midpoint of the group. Finally, we digitized 273 paths. Due to the characteristics of shared space, the pedestrian paths were greatly influenced by not only driving vehicles but also parked vehicles. Hence, the trace mapping results were presented by dividing the time slot according to the presence or absence of parked vehicles.

We then analyzed the digitized trace mapping data using GIS and three indicators: “Curvature of walking path”, “proportion of walking trace in central part of the street”, and “informal crossing ratio”. “Curvature of walking path” is the actual walking distance divided by the length of street segment (i.e., shortest distance). Since curvature is influenced by street width, we also used “corrected curvature” which is the value of the curvature divided by the diagonal length of the segment. “Proportion of walking trace in central part of the street” means the proportion of the length of the walking trace included in the central part of the street among the total length of the walking trace. Here, the central part refers to the area excluding both edges of the road (“roadside zone”) after dividing the walkable area into four parts considering obstacles such as on-street parking (Figure 3). Lastly, “informal crossing ratio” is defined as the ratio of the total number of walking traces to the number of walking traces that pass through the centerline of the walkable area at least once. Consistent with the above indicator, it also considered obstacles when defining the centerline. We expected that the larger the value of the indicators explained so far, the more freely the pedestrians made use of the entire space on the street.

$$Curvature=\;\frac{Actual\;distance\;of\;walking}{The\;shortest\;distance\;\left(length\;of\;street\;segment\right)}$$

$$Corrected\;Curvature=\;\frac{Curvature}{Diagonal\;length\;of\;the\;street\;segment}$$

## 3. Results

### 3.1. Perceived Safety in PPSs by Design Type

#### 3.1.1. One-Way ANOVA and Ordered Logit Analysis Results

Table 4 shows one-way ANOVA results of the participants’ perceived safety by street type. The results suggest that pedestrians’ level of safety statistically differs by design type. *Post hoc* multiple comparison analysis using the Turkey method also demonstrated that the mean differences between all pairs were significant except pair B and C. This implies that the existence of roadside pedestrian zone paved by stamped ascon (i.e., visual separation) may affect pedestrians’ perceived safety level.

Table 5 shows the results of the ordered Logit regression analysis. As shown, all type of pavement design tends to be positively associated with perceived safety compared to the general back road. This means that the pavement design using stamped ascon technique in PPSs has a positive effect on reducing fear of traffic accidents in shared streets. However, in contrast to design intent of PPS, types B and C, with substantial or partial roadside pedestrian zones, show larger coefficients than type A. It implies that regardless of planner’s intention and project objective of PPS, the general public prefers shared streets with exclusive pedestrian zones. However, our regression model does not control for other street conditions including width, height of surrounding buildings, and traffic volume. To minimize this fundamental limitation of the model, we conduct a *post-hoc* in-depth interview in the following sub-section.

The other control variables also show reliable results. First, the participants who had accident experiences of acquaintances answered negatively about the perceived safety. Second, participants who believe that the current speed limit on back roads is adequate evaluated the safety level of the roads higher than those who think that the standard should be further strengthened. Lastly, participants who were majoring in urban/transportation studies or in higher year of study responded that the streets are more dangerous than those with other majors or lower years. It is believed that the greater the knowledge of related majors, the higher the standard for the street environment.

#### 3.1.2. *Post-Hoc* Interview Results

Among the participants, 23 volunteers were interviewed after the visual assessment survey. This interview mainly aimed to identify the key reasons why they felt the streets to be safe or dangerous. Table 6 summarizes the results of the interview content analysis focusing on the frequently mentioned key words. They suggested seven main opinions regarding three main key words: road width, roadside pedestrian zone, and integrated design of road surface.

Regarding the road width, 14 interviewees mentioned that the width of the given streets influenced the risk perception. In particular, most of them said that the Gwanak-ro 14-gil (type A) seemed to be less safe due to its narrow width. Even though we set the same distance between the passing vehicle and the camera for all streets to minimize bias from street width in our experiment, the size of the visually empty space seemed to greatly influence the participants’ safety awareness. In addition, 10 interviewees responded that integrated surface designs like type A could create a pedestrian-friendly environment. Thus, the relatively low preference for the Gwanak-ro 14-gil (type A) might be attributed to the narrow width, not the pavement design.

With regard to roadside pedestrian zones, the interviewees offered conflicting opinions consistent with a discrepancy between design intent and user demand. 12 interviewees who were pro-roadside pedestrian zones said that they could feel a sense of psychological stability when they were guaranteed an independent pedestrian area. Likewise, Kaparias et al. argued that the introduction of a certain portion of pedestrian zones on shared streets plays a role in allowing pedestrians to use the entire road more comfortably [29]. In other words, a positive perception of the safety provided by the designated pedestrian zone can affect the safety perception of the whole street. On the contrary, 9 interviewees responded that the more clearly the pedestrian zone is distinguished on the shared street the more dangerous it can be to pedestrians. The anti-roadside pedestrian zone primarily concerned the increase in vehicle speed in exclusive driving spaces. If pedestrians do not have to go outside the pedestrian zone, this type of street may feel safer. However, pedestrians may feel a greater threat from vehicles in the exclusive vehicle space if pedestrians have to use the central part of the street because of parked vehicles or obstacles at the roadside pedestrian zone. Lee and Kim showed that the driving speed has rather increased in the case of PPS project applying a C-type design and argued that it is difficult to expect substantial improvement of safety as the design aiming to secure exclusive pedestrian space provides exclusive driving space at the same time [1].

### 3.2. Free Walking in PPSs by Design Type

#### 3.2.1. Results of Pedestrian Movement Tracing and Analysis by Design Type

The trace mapping results for the four streets and nine time slots are shown in Table 7. The results demonstrate that walking along the edge of the road is dominant movement pattern except in type A. To analyze pedestrians’ degree of free walking (unconstrained walking) in PPSs by design type in detail, we quantitatively measured the patterns using three indicators explained in Section 3: “Corrected curvature”, “proportion of walking and trace in central part of the street”, and “informal crossing ratio”.

As shown in Table 8, the degree of free walking was the highest in the type A street in terms of three indicators, whereas it was the least effective in improving the sense of safety among the three design types. As explained throughout the paper, the type A street best followed the design principles of the PPS project of minimizing the feeling of a vehicle-oriented space by applying a pavement design that allows the entire road space to be recognized as a single space without dividing the areas between pedestrians and vehicles. Therefore, the result can be regarded as the evidence proving the effectiveness of the PPS project. In contrast, types B and C did not show statistically significant differences from the control street. It can be interpreted as showing that the design of stamped asphalt pavement, which alludes to a roadside pedestrian zone, limited the free movement of pedestrians throughout the road. In particular, type C showed lower mean corrected curvature of pedestrian movement than that of control street. It means that if pedestrian and vehicle areas are completely distinguished through the design pavement in the PPS (in the form of a flush surface), the free movement of pedestrians is restricted compared to ordinary back roads on which the project has not been implemented.

#### 3.2.2. Stratified Analysis Considering On-Street Parking in PPS

Due to the nature of the shared space, the pedestrian path is greatly influenced not only by driving vehicles but also by parked vehicles. In consideration of this, a stratified analysis was conducted by separating the videos based on the time when the number of parked vehicles changed. Table 7 and Table 9 demonstrate pedestrian movement trace mapping and analysis result by number of parked vehicles. According to the three indicators, pedestrians seemed to use the entire street more freely where there are parked vehicles. In particular, all streets showed higher informal crossing ratio if there were more parked cars. In this case, ostensibly, the results seem to correspond well with the purpose of the PPS project, but in reality, it should not be regarded as freer and safer behaviors because pedestrians are forcibly pushed toward the center of the road rather than doing so voluntarily. This result indicates that the degree of change in the path of pedestrians by parked vehicles is greater than expected since the positions of the roadside zone and the center line are adjusted to the range in which pedestrians can move (see Figure 3). In the case of type C, since an almost exclusive driving space was provided in the middle of the road, this kind of walking pattern can be considered very vulnerable in terms of pedestrian safety.

## 4. Conclusions

To address hostile walking condition in back roads, the Seoul city government has carried out PPS projects that are mainly aimed to encourage “safe” and “convenient” walking of pedestrians. This study has investigated these two main goals by conducting a visual assessment survey with recorded VR video, *post-hoc* interview, and trace mapping and analysis.

With respect to the perceived safety level of pedestrians, the ordered Logit analysis results showed that the participants perceived higher safety levels in three PPSs applying distinctive pavement design than in general back road (control street). This is evidence that even small changes in road surface design can make pedestrians feel safer in shared space. However, the coefficient size varied depending on the pavement design type. Unlike our expectations and the design intent of PPS project, the participants felt higher safety levels in types B and C than in type A. When interpreted together with the *post-hoc* interview results, this seems to indicate that the participants felt greater influence from the width of the road than the type of surface design of each road. Opinions were divided on the appropriateness of suggesting a roadside pedestrian zone with stamped asphalt pavement. There were slightly more respondents of the opinions that it would be safer for the pedestrian area to be clearly separated from the vehicle area as in Type C (12 people), but concerns that vehicle speeds would increase further in the exclusive driving area and that the pedestrian area without a raised sidewalk could be rather vulnerable to vehicle intrusion were expressed (9 people). Therefore, in terms of perceive safety, it is still difficult to conclude which type of pavement design is more desirable.

Regarding the convenience of walking, the trace mapping and analysis result demonstrated that the degree of free walking was the highest in the type A street as anticipated in terms of “corrected curvature”, “proportion of walking and trace in central part of the street”, and “informal crossing ratio”. The type A street best followed the design principles of PPS project. Stamped asphalt pavement covered the entire street and there were no suggestions at the roadside of an exclusive pedestrian zone. On the other hand, compared to ordinary back roads that have not implemented the project, pedestrians’ free movement was more restricted in type C, which had clearly separated spaces through pavement surface design. Thus, in order to induce behavioral changes that would allow pedestrians to move more freely in a shared street, it is necessary to follow the design principle of PPS, which does not distinguish between pedestrians and vehicle areas, as in type A. However, since pedestrians’ trust in the safety of PPSs—which have not been distinguished in pedestrian areas—is not yet well-grounded, it is necessary to consider measures to enhance safety through reinforcement of speed limits.

Unlike previous studies that focused on vehicle speed and traffic accidents, this study is meaningful in that it verified the effectiveness of the PPS project by analyzing pedestrians’ movement trajectories in shared spaces as well as actual road user’s perspective such as perceived safety, which are rarely investigated. These results could expand the discussion in conjunction with existing studies about actual safety, and additional research is required to examine the gap between risk perception and actual danger of pedestrian. In terms of methodology, this study also has several benefits over previous studies. First, thanks to the extensive merits of recorded VR video and HMD, we can safely implement and film a dangerous situation that is often seen on shared streets, and allow many participants to experience the situation safely, conveniently and realistically without going directly to the street. Whereas simulated VR has been widely used as a risk-free safety research and education tool in the field of urban and transportation planning [30,31,32], recorded VR has rarely been used until recently. Moreover, many studies still employ semi-immersive VR environments in their visual assessment survey [31,33,34,35]. Second, this study applied both qualitative and quantitative research approaches. Using “mixed-method” allowed the respective results to complement each other and afforded us new insights to understand them [36]. By employing a *post-hoc* interview, we tried to minimize the limitations of our VR experiment and econometric analysis.

This study has several shortcomings. First, since our experimental conditions were directly implemented in real-world urban spaces, not simulated VR studies utilizing virtual spaces, it was difficult to design completely controlled experiments. Accordingly, we could not control for many environmental factors such as street width and surrounding building characteristics that might affect safety/risk perception in a street. Even though we set the same distance between the passing vehicle and the camera for all streets to minimize bias from different street width, the width has been shown to have a significant impact on perceived safety. In addition, in order to control for the influence of the number of vehicles and pedestrians, the number of passing vehicles was limited to one vehicle driven by the author, and filming was conducted on a weekend morning to avoid pedestrians whose movements were difficult to restrict. However, these experimental conditions are clearly distinctive from the usual back road conditions in Seoul, which are crowded with many vehicles and pedestrians.

Second, in this study, only one street was selected and analyzed for each design type. We tried to select streets that were representative of each type and had relatively similar conditions besides the pavement design. However, as the number of cases was small, there was a limitation in analyzing more diverse cases. Therefore, future studies are required to reconfirm our findings by adding new study areas where the project has recently been conducted, or use simulated VR to conduct analysis under fully controlled conditions other than pavement design.

Furthermore, like other research using VR [37], this study targeted a limited population cohort of university students and only 50 students finally participated in our survey through snowball sampling, thus making generalizing our results difficult. To expand our study, future research should cover a wide range of people, including children, the elderly, and the disabled. Although our target population and sample size are not very different from those of previous VR studies [37], this study should be regarded as a pioneering study applying novel methodology, rather than a confirmatory study.

Lastly, it should be noted the walking behavioral characteristics found in this study may vary depending on the season and weather. However, in order to meet these conditions equally among the target streets, filming was conducted simultaneously (i.e., at the same time on the same day) in typical autumn weather, which is generally suitable for outdoor activities in Korea.

## Figures and Tables

**Table 1:** The type of paving design by the extent of “visual separation (VS)”.

Type	Explanation
**A**	Stamped asphalt pavement covered the entire width of the street and there were no suggestions at the roadside of an exclusive pedestrian zone. This concept was interpreted to intend a genuine coexistence of pedestrians and vehicles.
**B**	Stamped asphalt pavement covered the entire width of the street, and there was some suggestion at the roadside of a pedestrian zone. This was interpreted as intending to protect a minimum area for pedestrians, while pursuing user coexistence.
**C**	Stamped asphalt pavement covered just a part of the street, which implied that pedestrians should walk within the paved area. This was interpreted as not pursuing coexistence.

Adapted from Lee and Kim [1] (pp. 6–7).

**Table 2:** Details of the study sites.

Type	Street Design	Site Information
**A**	Gwanak-ro 14-gil	
	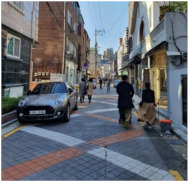	∙ Project year: 2017 ∙ Width: 6 m ∙ Total length: 430 m ∙ Traffic volume per hour: 64 ∙ Pedestrian volume per hour: 629
**B**	Baekjegobun-ro 7-gil	
	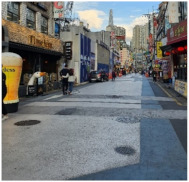	∙ Project year: 2016 ∙ Width: 8 m ∙ Total length: 500 m ∙ Traffic volume per hour: 192 ∙ Pedestrian volume per hour: 451
**C**	Godeok-ro 38-gil	
	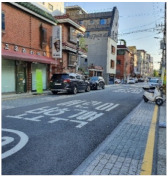	∙ Project year: 2014 ∙ Width: 10 m ∙ Total length: 710 m ∙ Traffic volume per hour: 136 ∙ Pedestrian volume per hour: 234
**Control group**	Cheongnyong-gil	
	** 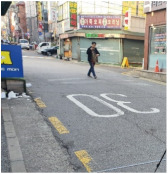 **	∙ Width: 7 m ∙ Traffic volume per hour: 270 ∙ Pedestrian volume per hour: 238

**Table 3:** Basic characteristics of the participants.

Variables	Frequency						Mean	Std Dev
	*1 (Rarely)*		*2 (Sometimes)*		*3 (Usually)*			
***Frequency of walking on a typical shared street in a week ^1^***	11		10		29		2.36	0.83
***Frequency of walking on a PPS in a week ^1^***	42		7		1		1.18	0.44
	***0 (no)***			***1 (yes)***				
***Driving*** *(Whether you can drive)*	26			24			0.48	0.50
***Own vehicle*** *(Whether you have your own car)*	48			2			0.04	0.29
***Accident 1*** *(Whether you have been in a car accident or crash when walking)*	41			9			0.18	0.39
***Accident 2*** *(Whether your family or friends have been in a car crash or accident when walking)*	37			13			0.26	0.44
***Speed limit desirability*** *(Whether you think the current speed limits (30 m/h) on a shared street is desirable)*	19			31			1.38	0.49
***VR HMD experience*** *(Whether you have used VR headsets and equipment)*	17			33			0.66	0.48
***Relevant Major*** *(Whether your major is related to Urban Engineering or Traffic Engineering)*	13			37			1.62	0.88
	***1***	***2***	***3***	***4***	***5***	***6***		
***Years of education*** *(from freshman)*	2	1	20	16	7	4	4.68	1.24

^1^ Rarely: 0–2 days, Sometimes: 3–4 days, Usually: 5–7 days; number of observation = 50.

**Table 4:** One-way ANOVA results for participants’ perceived safety.

Design Type	Mean	Std Dev	df	F
Type A	3.68	1.22	Between Groups = 3 Within Groups = 196	40.76 ***
Type B	4.88	1.12		
Type C	4.96	1.29		
Control street	2.70	1.13		
Total	4.06	1.51	199	

*** = *p* < 0.01.

**Table 5:** Ordered Logit regression model of perceived safety.

Variable		B	Std. Error	Wald	Sig
*Type: “Control street” is reference variable.*					
Pavement design type	A	1.672	0.391	18.277	0.000 ***
	B	3.468	0.435	63.426	0.000 ***
	C	3.678	0.441	69.446	0.000 *
*Frequency of walking: “Usually” is reference variable.*					
Frequency of walking on a typical shared street	Rarely	0.439	0.344	1.633	0.201
	Sometimes	0.521	0.367	2.009	0.156
Frequency of walking on a PPS	Rarely	−0.065	0.977	0.004	0.947
	Sometimes	0.161	1.032	0.025	0.876
Driving		−0.209	0.278	0.568	0.451
Own vehicle		1.122	0.707	2.521	0.112
Accident 1 (himself/herself)		0.336	0.352	0.909	0.340
Accident 2 (family/friends)		−0.595	0.341	3.039	0.081 *
Desirability of speed limit		0.624	0.305	4.191	0.041 **
VR HMD experience		0.052	0.307	0.029	0.865
Relevant major		−1.107	0.347	10.155	0.001 ***
Years of education		−0.324	0.125	6.743	0.009 ***

* *p* < 0.10, ** *p* < 0.05, *** *p* < 0.01; Pseudo R^2^: Cox & Snell (0.439), Nagelkerke (0.452); *n* = 200.

**Table 6:** Results of interview content analysis.

Categories	Condensed Meaning Unit (Counts)	Meaning Unit (Counts)
Road width	▪ The width of the given streets influenced the evaluation. (14)	“*In the case of type A, the road was narrower than the other types and felt more dangerous*.” (7)
		“*In the case of type A, if the width of the sample street was wider, it would have been more positive*.” (4)
		“*In the case of type B and type C, the road was wide, so I felt safe*.” (3)
	▪ The width of the street did not have much effect on the judgment. (1)	“*Since we know that the narrower the road, the more carefully the driver drive, so the road width did not significantly affect the evaluation*.” (1)
	▪ The desired PPS design varies depending on the width of the road. (6)	“*It is most desirable to apply type A to narrow streets*.” (5)
		“*As with the examples of type B and type C, on wide roads, designs with pedestrian zones are better than those without*.” (1)
Roadside pedestrian zone	▪ It feels safe when the pedestrian zones are clearly separated. (12)	“*When an area where pedestrian can exclusively pass is determined, it is comfortable because it is free inside the space*.” (7)
		“*Even if the pedestrian area existed on the roadside, as a pedestrian, I can use the entire road freely, and if there is a pedestrian area, it seems that I can easily avoid dangerous situations*.” (2)
		“*When the pedestrian area is separated, it is likely that the drivers are at least careful not to invade the zone*.” (3)
	▪ In the shared street, the more clearly the pedestrian area is separated, the more dangerous it is. (9)	“*In the typical shared street, the clearer the pedestrian zone, the more dangerous it will be that the driver will speed up without paying attention to the pedestrian*.” (5)
		“*Pedestrian zones with level surface like type C are perceived as a more dangerous situation because there is room for vehicle invasion and it will be used as a street parking space*.” (4)
Integrated design of road surface	▪ Integrated design (type A) creates a pedestrian-friendly environment. (10)	“*Type A looks like a pedestrian path, so it feels unfamiliar for vehicles to pass by*.” (5)
		“*Even if the pedestrian area is distinguished, if the paving design of type A is actively used for the entire width of the road, the street can be used more comfortably for pedestrians*.” (2)
		“*In the street with a type A design, I expected the vehicle to slow down*.” (3)
Others	▪ The desired PPS design varies depending on traffic or pedestrian volume. (1)	“*The same design is thought to work differently depending on pedestrian or traffic volume on the street, and I think the design should be introduced differently according to the conditions*.” (1)

**Table 7:** Trace mapping results by design type and time slot (number of parked vehicles).

**Type A**	A-1 (13:30–13:34, 0 parked car)	A-2 (13:34–13:35, 2 parked cars)
	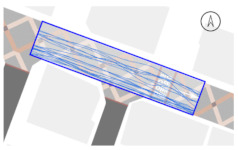	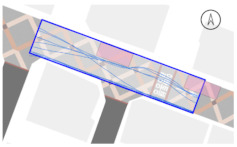
	A-3 (13:35–13:38, 1 parked car)	A-4 (13:38–13:45, 0 parked car)
	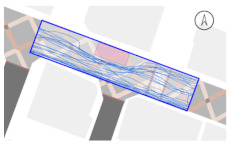	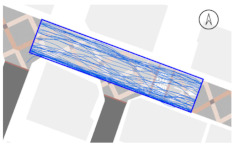
**Type B**	B-1 (13:30–13:34, 1 parked car)	B-2 (13:34–13:45, 0 parked car)
	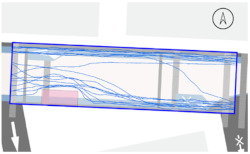	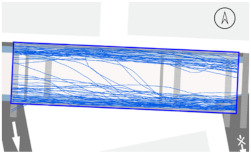
**Type C**	C-1 (13:30–13:32, 4 parked cars)	C-2 (13:32–13:45, 3 parked cars)
	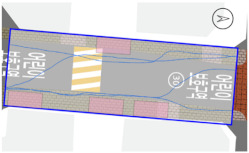	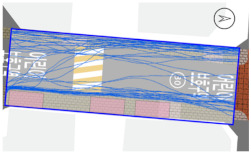
	Control group (13:30–13:45, 1 parked car)	
**Ctrl. street**	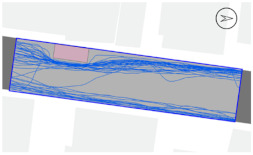	

**Table 8:** Comparison of degrees of free walking in Pedestrian Priority Streets (PPSs) by design type.

Type	*n*	Mean Corrected Curvature	Mean Proportion of Walking Trace in Central Part of the Street (%)	Informal Crossing Ratio (%)
A	92	0.040 (56.086) ***	35.97 (4.478) ***	29.35
B	86	0.033 (−0.058)	13.39 (−0.883)	12.79
C	50	0.032 (−6.109) ***	21.84 (1.420)	18.00
Control street	45	0.033	16.00	11.11

Note: With respect to the “corrected curvature” and “proportion of walking trace in central part of the street”, we compared mean differences between each-type of street and the control street using *t*-tests (*t*-values are shown in parenthesis). *** *p* < 0.01.

**Table 9:** Movement patterns by design type and time slot (number of parked vehicles).

Type	Time Slot	*n*	Number of Parked Cars	Mean Corrected Curvature	Mean Proportion of Walking Trace in Central Part of the Street (%)	Informal Crossing Ratio (%)
A	1	19	0	0.0395	48.01	26.32
	2	8	2	0.0396	37.71	50.00
	3	20	1	0.0400	41.36	50.00
	4	45	0	0.0396	28.19	17.78
	Total	92	-	0.0397	35.97	29.35
B	1	18	1	0.0328	15.70	27.78
	2	68	0	0.0331	12.79	8.82
	Total	86	-	0.0330	13.39	12.79
C	1	4	4	0.0327	27.15	50.00
	2	46	3	0.0321	21.37	15.22
	Total	50	-	0.0322	21.84	18.00
Ctrl. street	1	45	1	0.0330	16.00	11.11
